# Evaluation of models to predict prognosis in patients with advanced hepatocellular carcinoma treated with TACE combined with apatinib

**DOI:** 10.1186/s12876-024-03210-1

**Published:** 2024-04-08

**Authors:** Fang Sun, Kai-Cai Liu, Qurat Ul Ain, Dong Lu, Chun-Ze Zhou, Jing-Kun Xiao, Xing-Ming Zhang, Zheng-Feng Zhang, Deng-Lei Cheng, Yu-Sheng He, Wei-Fu Lv

**Affiliations:** 1 Department of Radiology, Anhui Provincial Hospital of Anhui Medical University, Hefei, China; 2 Infection Hospital(Hefei Infectious Disease Hospital), The First Affiliated Hospital of USTC, Division of Life Sciences and Medicine, University of Science and Technology of China, Hefei, China; 3 The First Affiliated Hospital of University of Science and Technology of China, Division of Life Sciences and Medicine, University of Science and Technology of China, Hefei, China China; 4 Department of Radiology, The First Affiliated Hospital of University of Science and Technology of China (USTC), Division of Life Sciences and Medicine, University of Science and Technology of China, 230000 Hefei, China

**Keywords:** Hepatocellular carcinoma, Transarterial chemoembolization, Apatinib, Model, Prognosis, Overall survival

## Abstract

**Background:**

The HAP, Six-and-Twelve, Up to Seven, and ALBI scores have been substantiated as reliable prognostic markers in patients presenting with intermediate and advanced hepatocellular carcinoma (HCC) undergoing transarterial chemoembolization (TACE) treatment. Given this premise, our research aims to assess the predictive efficacy of these models in patients with intermediate and advanced HCC receiving a combination of TACE and Apatinib. Additionally, we have conducted a meticulous comparative analysis of these four scoring systems to discern their respective predictive capacities and efficacies in combined therapy.

**Methods:**

Performing a retrospective analysis on the clinical data from 200 patients with intermediate and advanced HCC, we studied those who received TACE combined with Apatinib at the First Affiliated Hospital of the University of Science and Technology of China between June 2018 and December 2022. To identify the factors affecting survival, the study performed univariate and multivariate Cox regression analyses, with calculations of four different scores: HAP, Six-and-Twelve, Up to Seven, and ALBI. Lastly, Harrell’s C-index was employed to compare the prognostic abilities of these scores.

**Results:**

Cox proportional hazards model results revealed that the ALBI score, presence of portal vein tumor thrombus (PVTT, )and tumor size are independent determinants of prognostic survival. The Kaplan-Meier analyses showed significant differences in survival rates among patients classified by the HAP, Six-and-Twelve, Up to Seven, and ALBI scoring methods. Of the evaluated systems, the HAP scoring demonstrated greater prognostic precision, with a Harrell’s C-index of 0.742, surpassing the alternative models (*P* < 0.05). In addition, an analysis of the area under the AU-ROC curve confirms the remarkable superiority of the HAP score in predicting short-term survival outcomes.

**Conclusion:**

Our study confirms the predictive value of HAP, Six-and-Twelve, Up to Seven, and ALBI scores in intermediate to advanced Hepatocellular Carcinoma (HCC) patients receiving combined Transarterial Chemoembolization (TACE) and Apatinib therapy. Notably, the HAP model excels in predicting outcomes for this specific HCC subgroup.

## Introduction

Liver cancer is the fifth most prevalent form of cancer globally and the second most frequent contributor to cancer-related fatalities, highlighting its substantial implications for public health. Hepatocellular Carcinoma (HCC) dominates primary liver cancers, accounting for approximately 90% of cases, underscoring the critical need for the international community to prioritize the fight against liver cancer [1]. Risk factors for HCC include chronic viral hepatitis infections (types B and C), alcoholic liver cirrhosis, and exposure to aflatoxins. Notably, with the hepatitis B virus being highly prevalent in China, over 80% of HCC patients in the country have a history of this viral infection [2]. HCC has an insidious onset and is highly malignant. Most patients receive a diagnosis in advanced stages, leading to a bleak prognosis [3]. Transarterial chemoembolization (TACE) is currently the preferred local therapy for intermediate to advanced phases of HCC [1, 4, 5]. TACE is a procedure that involves administering chemotherapy drugs directly into the arteries supplying the tumor, along with embolic agents. This method elicits cytotoxic reactions in the tumor and obstructs blood supply to the tumor tissue, impeding the advancement of the tumor. However, research has demonstrated that following a TACE procedure, the vascular endothelial growth factor (VEGF) expression within blood vessels in the tumor significantly rises. This increase in VEGF triggers heightened vascular activity, leading to a concurrent enhancement of residual tumor vascularization and collateral circulation [6, 7].. Vascular endothelial growth factor receptor 2 (VEGFR-2) is a crucial element in the process of angiogenesis, binding to VEGF and serving as an essential target in anti-angiogenic therapy. Consequently, a combination therapy using targeted anti-angiogenic agents has surfaced as a critical treatment regimen [6, 8]. Vascular endothelial growth factor receptors (VEGFRs) belong to the receptor tyrosine kinase (RTKs) family. Apatinib is a potent, orally administered, highly selective tyrosine kinase inhibitor that targets specifically VEGFR-2. By inhibiting endothelial cell proliferation, it suppresses tumor growth. Apatinib has recently received approval from the National Medical Products Administration (NMPA) as a second-line therapy for patients with liver cancer [9]. Research has revealed that the combination of TACE and Apatinib offers improved long-term outcomes for intermediate to advanced HCC compared to TACE monotherapy. Thus, it presents an alternative therapeutic option for advanced HCC patients [10]. The available treatments for HCC are numerous due to its heterogeneity, and the prognosis remains uncertain. TACE and Apatinib have shown improved survival rates and quality of life for advanced stage HCC patients. However, it is unclear which patients would benefit from this treatment [11]. Therefore, the need for a staging system to predict the prognosis of HCC patients undergoing TACE with Apatinib treatment is urgent. This will aid in choosing the appropriate patient for selected treatment options.

Tumor size and number are crucial factors in determining treatment strategies within clinical settings. As a result, scoring systems have been developed to gauge tumor burden. Two examples of such systems are the Six-and-Twelve score and Up to Seven score. In addition, the Albumin-Bilirubin (ALBI) score, established early on, is used to evaluate liver function in HCC patients and is based on complex calculations using serum albumin and bilirubin levels. The HAP score also incorporates four variables related to tumors: alpha-fetoprotein, tumor size, serum albumin, and total bilirubin. These scoring systems have been validated to ascertain their effectiveness in predicting the prognosis of patients who have undergone TACE treatment [4, 12–14]. However, no previous research has evaluated the predictive value of scoring systems in patients who undergo TACE along with Apatinib treatment. Hence, this study aims to validate the usefulness of these scores in predicting the prognosis of such patients and to compare their predictive abilities. The goal is to enhance the accuracy of treatment plans and improve the assessment of patient prognosis.

## Methods

### Patient population

**Fig. 1:**
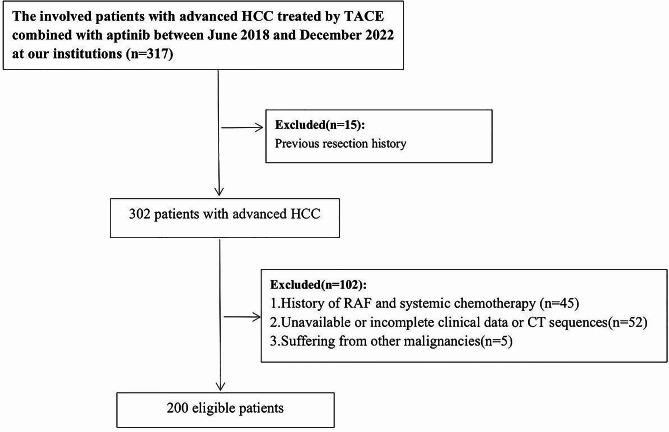
Flow chart for patient eligibility

We analyzed the clinical case data of 317 patients with intermediate and advanced HCC treated with TACE combined with Apatinib from June 2018 to December 2022 in our hospital. The inclusion criteria were as follows: (1) Confirmed diagnosis of HCC through pathological examination or based on the American Association for the Study of Liver Diseases (AASLD) practice guidelines; (2) Advanced HCC not suitable for liver resection or challenging to treat with other local treatments (such as radiofrequency ablation (RFA), percutaneous ethanol injection (PEI), and microwave ablation (MWA)); (3) ECOG score ≤ 2; (4) Child‒Pugh A or B; (5) At least one measurable lesion (> 10 mm) according to the modified Response Evaluation Criteria In Solid Tumors (mRECIST) for the efficacy of solid tumors; and (6) Continuously taking targeted drug therapy for ≥ 1 month. The exclusion criteria were as follows: (1) Child‒Pugh score>9; (2) severe hypersplenism with platelets less than 50 × 10^9^; (3) gastrointestinal bleeding experienced in the past 3 months; (4) presence of other primary malignant tumors; (5) severe heart, lung, or kidney dysfunction; and (6) incomplete follow-up data. The retrospective study was conducted following the principles of the Declaration of Helsinki. Ultimately, 200 patients were included in this study, and the process of the study is depicted in Fig. 1. The study protocol was approved by the ethics committee of the First Affiliated Hospital of University of Science and Technology of China(USTC), and written informed consent was waived for this retrospective study.

### Treatments

TACE was performed by experienced interventional radiologists who had at least 5 years of experience in TACE. The modifed Seldinger technique was adopted to puncture the right femoral artery, and then a 5Fangiography catheter was inserted into the common hepatic artery. After digital subtraction angiography (DSA), the size of the tumor, the blood supply artery of the tumor, the existence of hepatic artery hepatic vein fistula, and the abundance of blood supply were further evaluated, and then a 2.7 F-microcatheter was inserted into the target vessel of the tumor, and embolization was performed with 60–80 mg adriamycin or other chemotherapeutic drugs and 10–20 mL iodized oil as the mixed emulsifier. The dose of iodized oil usually needed to be determined in combination with the size of the lesion, and the maximum dosage usually did not exceed 30 mL. After embolization, the femoral artery was pressurized for 24 h. The ECG was monitored 8 h after the operation, and symptomatic treatment was given to protect liver function, to protect stomach, and to stop vomiting. The number of repeated TACE treatments was usually determined by the clinician according to the patient’s tumor control and liver function.

Commencing three days post-TACE, patients initiate oral administration of Apatinib for antitumor therapy, prescribed at a dosage of 500 mg per day, with 4 weeks designated as a treatment course. The dosage is modulated in accordance with the patient’s adverse reaction tolerance; patients may sustain the original dosage in the presence of minor adverse reactions. If an adverse reaction becomes intolerable, the dosage may be reduced to 250 mg per day or temporarily stopped. After the disappearance of adverse effects, gradually resume the original dose and continue to use the drug. If the duration of discontinuation exceeds 1 month, the patient was excluded.

### Follow up

Post-treatment, laboratory test indices are evaluated at 4–6 weeks intervals, encompassing routine blood and urine analyses, liver and kidney function tests, coagulation function, and alpha-fetoprotein levels. Imaging assessments, utilizing enhanced CT or MRI, are executed at weeks 4 and 8 post-treatment and subsequently every 8 weeks thereafter. Repeat TACE procedures could be performed upon confirmation of persistent tumor activity or the emergence of new lesions. All patients undergo follow-up, with overall survival (OS) being defined as the interval from the initial TACE treatment to the date of death or the concluding follow-up visit. Patients were informed of the potential occurrence of treatment-emergent adverse events (AEs) at any time during treatment. Treatment-emergent AEs were assessed according to the National Cancer Institute Common Terminology Criteria for Adverse Events (version 5.0). Continuous surveillance was carefully performed throughout the treatment period.

### Statistical analysis

Continuous variables were presented as medians and interquartile ranges, assessed via Mann-Whitney U tests, while categorical variables were shown as frequencies and percentages and analyzed with either chi-square or Fisher’s exact tests. Stratification was performed based on the four scoring systems, with survival times calculated and survival curves generated using the Kaplan-Meier method. Differences in survival between strata were determined using the log-rank test. The performance of the staging systems was compared by using the consistency index (C-index), with a C-index of 0.5 indicating no predictive ability and a C-index of 1.0 indicating perfect predictive ability. Further, variables significantly linked with survival (*P* < 0.05) were analyzed in the multivariate model to identify predictors that influence OS. The ability to predict survival time at 6, 12, and 24 months was evaluated objectively by computing the area under the curve of the receiver operating characteristic (ROC). All statistical analyses were conducted using R, version 4.1.1, with two-tailed tests and a significance level of *P* < 0.05.

## Results

### Characteristics of patients

Two hundred patients were stratified using four distinct scoring systems. Baseline characteristics are presented in Table 1. More than half of the patients were under the age of 60, with male patients making up 162 cases (81%). A history of hepatitis was noted in 140 patients (70%), while 72 patients developed extrahepatic metastasis (36%). Regarding the Child-Pugh classification, 120 patients (60%) were categorized as A, and 80 patients (40%) fell under category B. At the time of diagnosis for HCC, The tumor diameter ranges from 1.8 cm to 12.6 cm, with most patients having a tumor diameter ≤ 7 cm (*n* = 124; 62%). More than three tumor nodules were found in more than half of the patients (*n* = 114; 57%). Additionally, the majority of patients (*n* = 119; 59.5%) exhibited PVTT classified as Type I-II. Other noteworthy aspects were that more than half of the patients had serum AFP levels less than or equal to 400 ng/mL (*n* = 116; 58%), around a quarter of patients (*n* = 50; 25%) exhibited an Eastern Cooperative Oncology Group (ECOG) performance status greater than 1. In addition, 83 patients (41.5%) were classified as Grade 1 as per the ALBI score, while 117 patients (58.5%) were categorized under ALBI Grade 2.

**Table 1:** Basic characteristics of 200 patients included in the analysis

Variable	N	(N)%
Age		
≤ 60Y	125	62.5
> 60Y	75	37.5
Sex(n%)		
Male	162	81.0
Female	38	19.0
Liver disease(n%)		
Yes	140	70.0
No	60	30.0
Metastases		
NO	128	64.0
YES	72	36.0
Child-Pugh grade(n%)		0.776
A	120	60.0
B	80	40.0
ALBI grade(n%)		
1	83	41.5
2	117	58.5
AFP (n%)		
≤ 400	116	58.0
> 400	84	42.0
Tumor (cm)		
≤ 7	124	62.0
> 7	76	38.0
Tumor number(n%)		
≤ 3	86	43.0
> 3	114	57.0
Ecog score (n%)		
0–1	150	75.0
2	50	25.0
PVTT(n%)		
I-II	119	59.5
III-IV	81	40.5

### Evaluation of staging systems

**Fig. 2:**
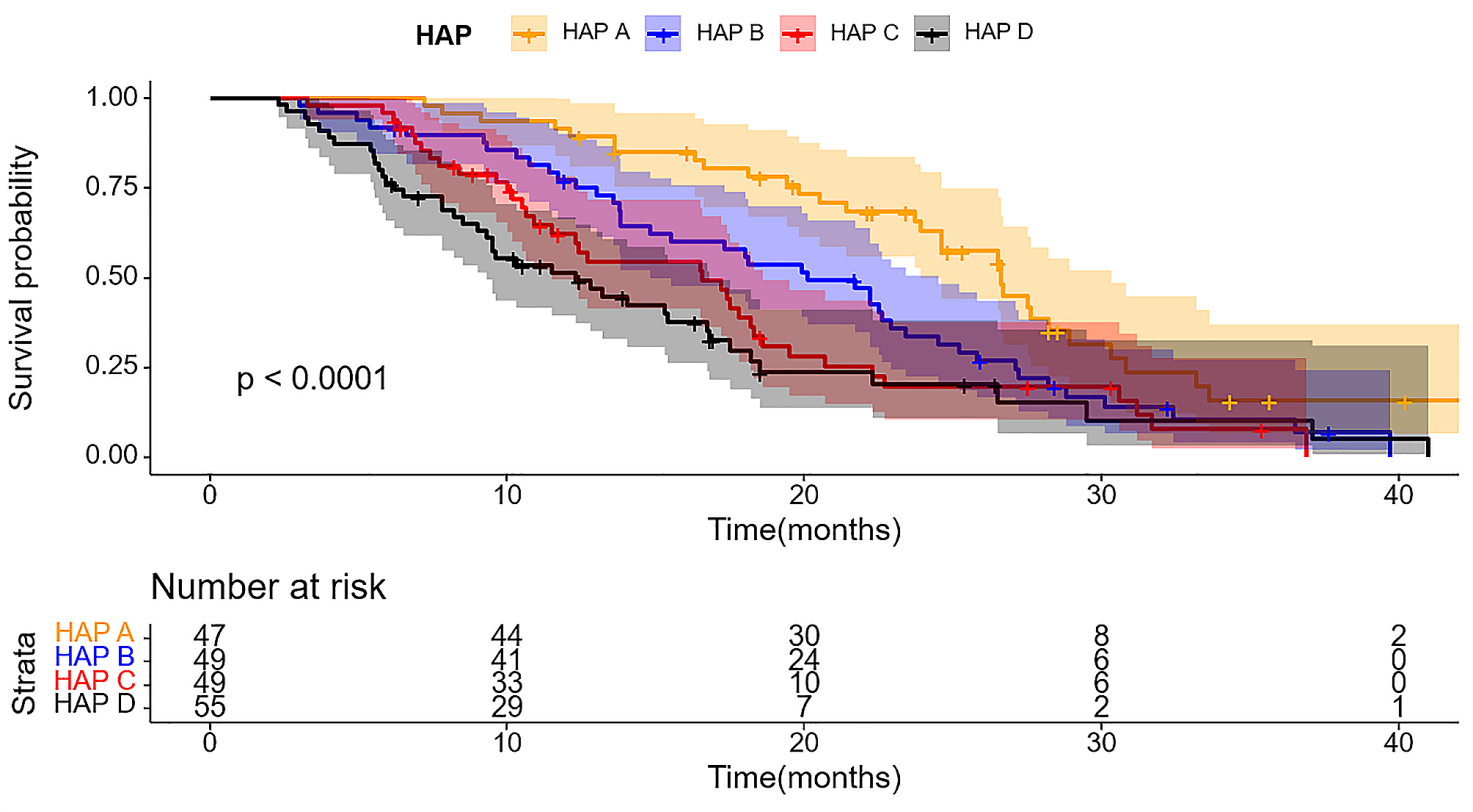
Kaplan-Meier curves illustrating overall survival (OS) in patients with hepatocellular carcinoma who underwent TACE and apatinib treatment stratified by HAP score

**Fig. 3:**
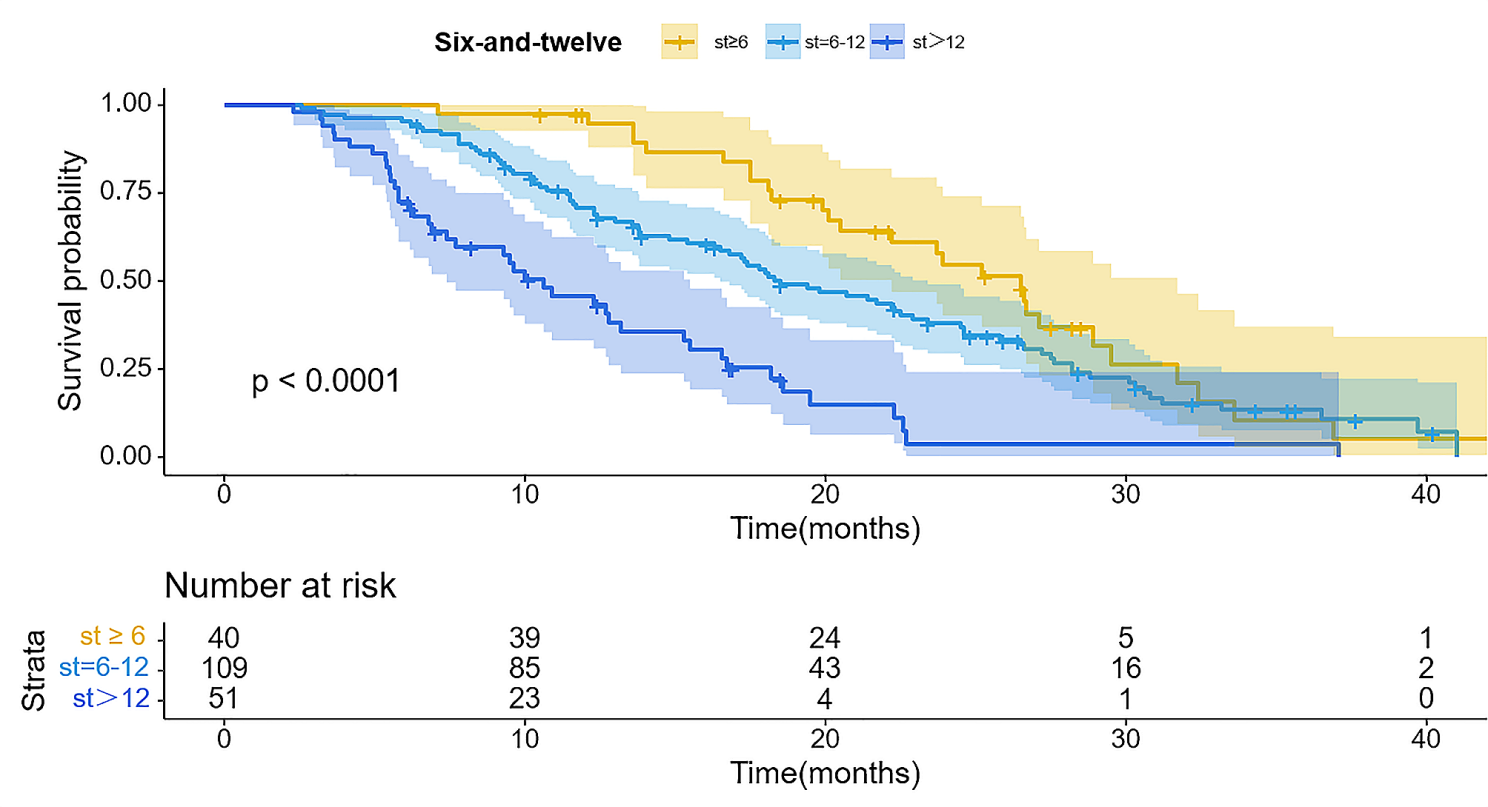
Kaplan-Meier curves illustrating overall survival (OS) in patients with hepatocellular carcinoma who underwent TACE and apatinib treatment stratified by Six-and-twelve score

**Fig. 4:**
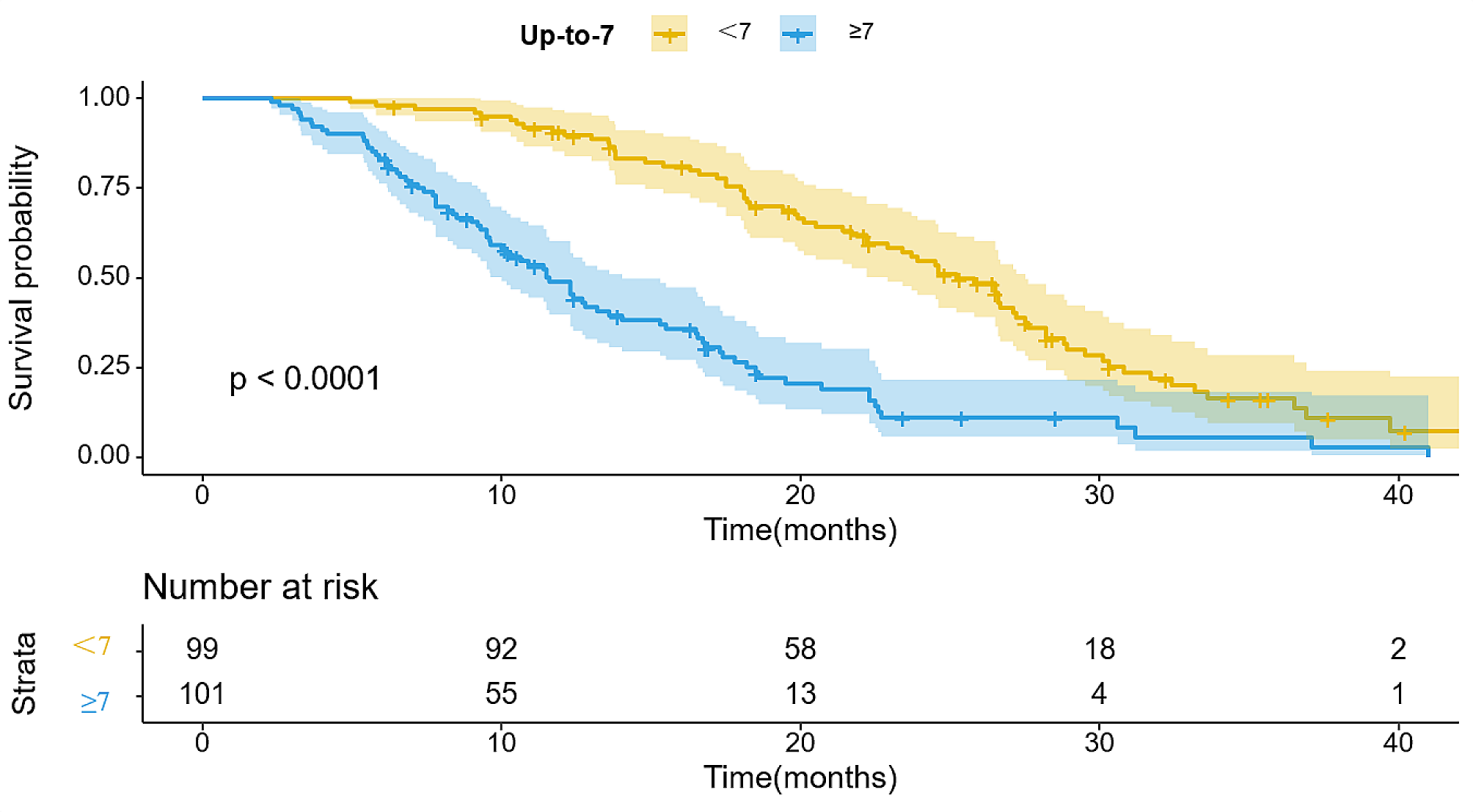
Kaplan-Meier curves illustrating overall survival (OS) in patients with hepatocellular carcinoma who underwent TACE and apatinib treatment stratified by Up-to-7 criterion

**Fig. 5:**
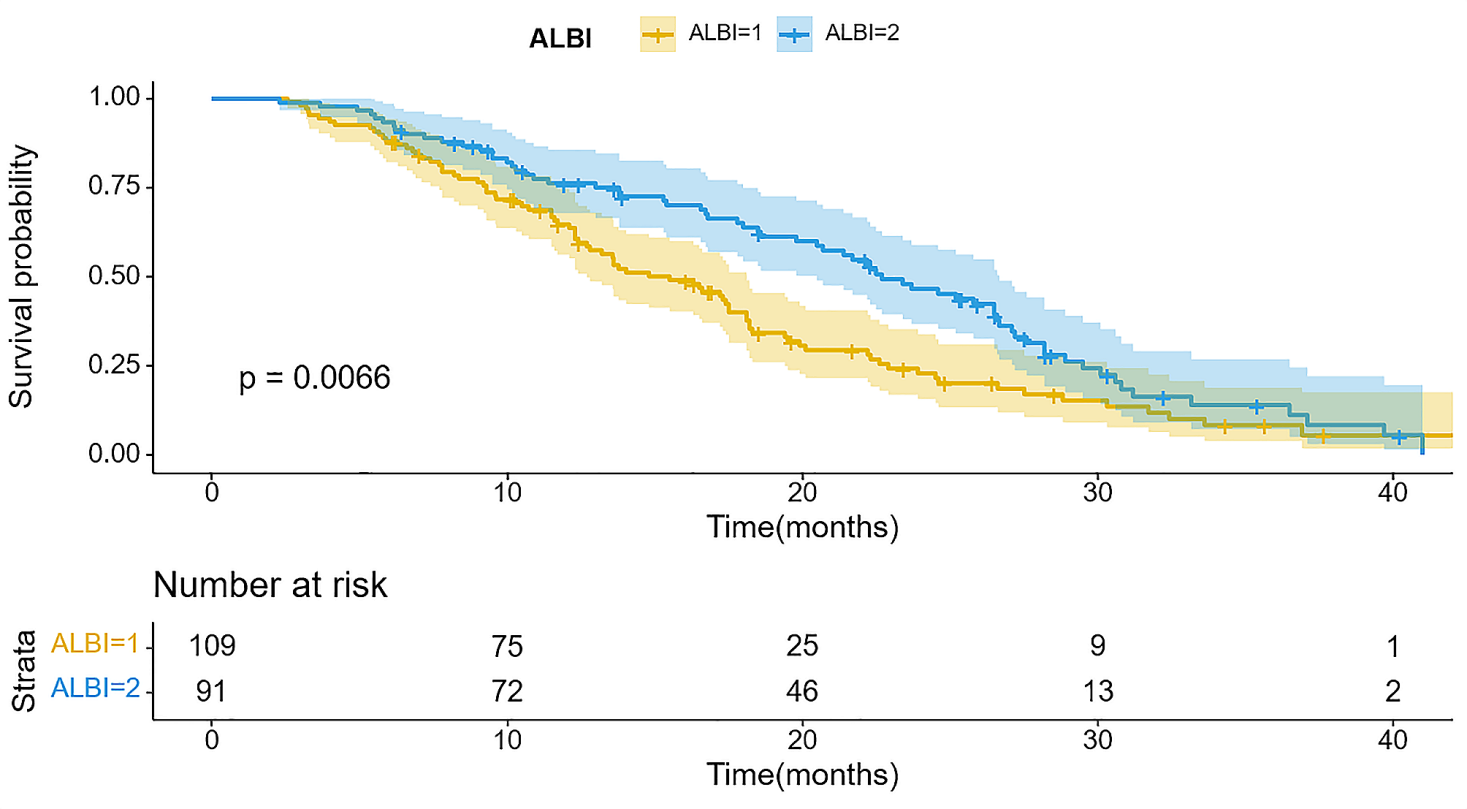
Kaplan-Meier curves illustrating overall survival (OS) in patients with hepatocellular carcinoma who underwent TACE and apatinib treatment stratified by ALBI grade

The wholestudy cohort displayed a median survival time (IQR) of 17.2 months. The importance of predictive value for survival duration was significant across four staging systems (*P* < 0.001), with comprehensive results in Table 2. Furthermore, all staging systems generated Kaplan-Meier curves, Using the HAP model, the stratification of groups A, B, C, and D demonstrated median survival of 23.1, 19.1, 14.9, and 12.5 months, respectively (Fig. 2). There was a significant difference in survival between the four groups (*P* < 0.001). According to the “six-and-twelve” grading system (≤ 6, > 6–12, > 12), median survival was 22.5, 18.3, and 10.7 months, respectively (*P* < 0.001) (Fig. 3). Within the “up to seven” grading system, patients with a tumor burden < 7 had significantly better median survival than those with a tumor burden ≥ 7 (22.3 vs. 12.3 months; *P* < 0.001) (Fig. 4). Similarly, patients with an ALBI grade of 1 had longer median survival than those with a grade of 2 (16.9 vs. 15.2 months; *P* < 0.001) (Fig. 5).

**Table 2:** Patient survival within each model based on log-rank test of KM

models	N	(N)%	OS
Total	200	100	17.2
HAP score			< 0.001
A	47	23.5	23.1
B	49	24.5	19.1
C	49	24.5	14.9
D	55	27.5	12.5
Six-to-twelve			< 0.001
≤ 6	40	20	22.5
6–12	109	54.5	18.3
> 12	51	25.5	10.7
Up to seven			< 0.001
< 7	99	49.5	22.3
≥ 7	101	50.5	12.3
ALBI grade			0.001
1	91	45.5	19.6
2	109	54.5	15.2

### Prognostic factors of OS

A univariate Cox survival hazard regression model analysis indicates that Child-Pugh classification (*P* < 0.05), ALBI score (*p* < 0.05), PVTT (*P* < 0.001), tumor size (*P* < 0.001), and AFP levels (*p* < 0.05) were significant predictive factors for patient survival, and were found to correlate with OS (Table 3). Subsequently, with *P* < 0.05, these five factors were included in a multivariate Cox survival hazard regression model analysis to determine the independent prognostic factors for OS. The study found that the ALBI score (Hazard Ratio (HR) = 0.624, 95% Confidence Interval (CI) 0.427–0.842, *p* = 0.012) had an independent effect on overall survival. Furthermore, the presence of PVTT (HR = 0.522, 95% CI 0.453–0.732, *p* < 0.001) and the size of the tumor (HR = 0.512, 95% CI 0.361–0.723, *p* < 0.001) were also identified as independent risk factors for overall survival. This data implies that ALBI score, PVTT, and tumor size might be more precise predictors of the survival time for HCC patients who receive TACE and Apatinib combination treatment (Table 3).

**Table 3:** Multivariable Cox Regression Analysis of Predictors of OS

Variables	Univariate Cox Regression		Multivariable Cox Regression	
	HR (95% CI)	P	HR (95% CI)	P
Sex(M vs. F)	0.659(0.434–1.003)	0.052		
Age (y)	0.998(0.984–1.013)	0.829		
Child-Pugh grade(A vs. B)	0.718(0.523–0.914)	0.038	0.827(0.625–1.139)	0.252
ALBI grade(1 vs. 2)	0.542(0.434–0.786)	0.001	0.624(0.427–0.842)	0.012
PVTT(I-II vs. III-IV)	0.455(0.343–0.631)	< 0.001	0.522(0.453–0.732)	< 0.001
Metastasis(Y vs. N)	0.877(0.675–1.234)	0.451		
Tumor diameter (< 7 cm vs. ≥ 7 cm)	0.429(0.325–0.631)	< 0.001	0.512(0.361–0.723)	< 0.001
Tumor number(< 3 vs. ≥ 3)	0.861(0.623–1.138)	0.345		
Hepatitis(Y vs. N)	0.951(0.684–1.326)	0.815		
ECOG(0–1 vs. 2)	1.312(0.722–1.421)	0.932		
AFP(< 400ng/ml vs. ≥ 400ng/ml)	0.574(0.370–0.851)	0.008	1.048(0.471–1.106)	0.102

### Comparing the performance of these scores

**Fig. 6:**
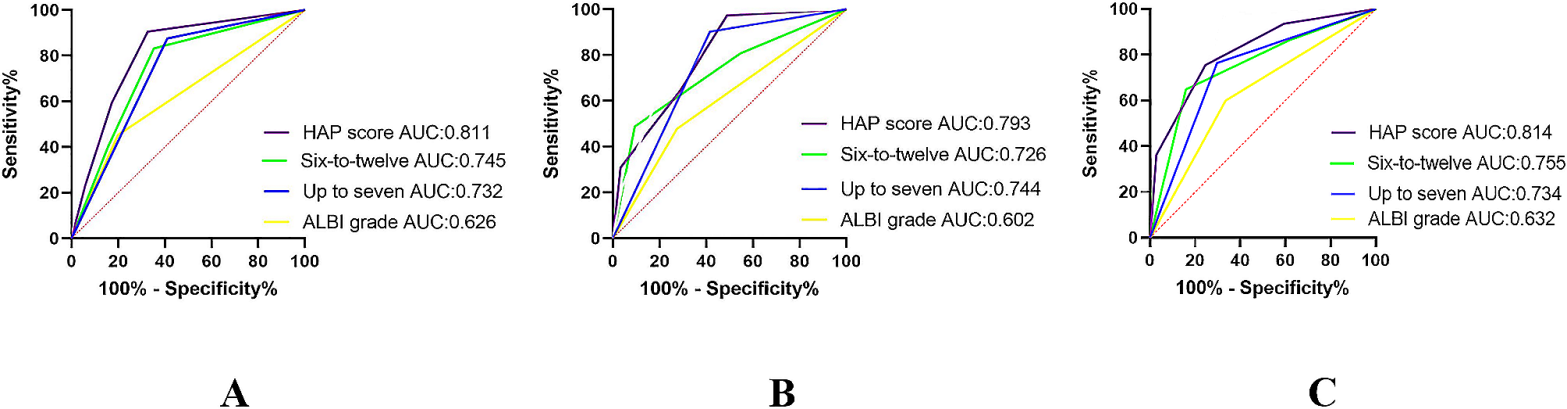
Time-dependent receiver operating characteristic analyses of HAP score, Six-and-twelve score, Up-to-7 criterion and ALBI grade for 6-month (**A**), 12-month (**B**) and 24-month (**C**) survival

We compared the prognostic capabilities of various staging systems using the C-index (Table 4). Among the four scoring systems, the HAP model exhibited the highest C-index at 0.742, indicating superior survival predictive ability, followed by the Six-and-Twelve score (0.701), Up to seven score (0.698), and ALBI score (0.601). Notably, the prognostic ability for survival of the HAP score was significantly superior to both the Up to seven score and the ALBI score (*P* < 0.001). Moreover, we utilized the Area Under the Receiver Operating Characteristic (AU-ROC) curve to quantitatively analyze the ability of the HAP score, Six-and-Twelve score, Up to seven score, and ALBI score in predicting OS at 6 months, 12 months, and 24 months. The results revealed that the 6-month AUC values for the HAP score, Six-and-Twelve score, Up to seven score, and ALBI score were 0.811, 0.745, 0.732, and 0.626, respectively; the 12-month AUC values were 0.793, 0.726, 0.744, and 0.602, respectively; and the 24-month AUC values were 0.814, 0.755, 0.734, and 0.632, respectively. The HAP score presented higher AUC values than the other three scores in predicting OS at 6 months, 12 months, and 24 months (Fig. 6(A-C)).

**Table 4:** Comparison of c-indices among models based on described statistical methods

model	C-index	95%CI		P
HAP score	0.742	0.744	0.853	-
Six-to-twelve	0.701	0.664	0.795	0.016
Up to seven	0.698	0.644	0.733	< 0.001
ALBI grade	0.601	0.531	0.712	< 0.001

### Safety profile

The details of treatment-related adverse events are delineated in Table 5, with hand-foot syndrome, hypertension, and oral mucositis emerging as the most prevalent adverse events. Notably, hand-foot syndrome exhibits the highest prevalence, encompassing various degrees of severity ranging from Grade 1 to Grade 4. Alopecia, on the other hand, shows relatively lower prevalence among patients, impacting only a limited number of individuals and not progressing to Grade 4 in terms of severity. Timely and symptomatic interventions were administered for all adverse reactions, and no fatalities related to adverse reactions were documented during the observation period.

**Table 5:** Adverse events in patients

Adverse events	patients (*n* = 200)			
	grade1(n%)	Grade2(n%)	Grade3(n%)	grade4(n%)
Hand and foot syndrome	45(22.50%)	38(19.00%)	32 (16.00%)	8(4.00%)
Hypertension	25(12.50%)	20(10.00%)	18 (9.00%)	7(3.50%)
Proteinuria	17(8.50%)	15(7.50%)	14 (7.00%)	3(1.50%)
Alopecia	5(2.50%)	4(2.00%)	1 (0.50%)	0
Fatigue	27(13.50%)	13(6.50%)	8 (4.00%)	1(0.50%)
Decreased appetite	23(11.50%)	11(5.50%)	14 (7.00%)	2(1.00%)
Diarrhea	15(7.50%)	11(5.50%)	3 (1.50%)	0
Oral mucositis	34(17.00%)	21(10.50%)	6 (3.00%)	1(0.50%)
hoarseness	6(3.00%)	4(2.00%)	7 (3.50%)	0
Abdominal pain	31(15.50%)	17(8.50%)	7 (3.50%)	1(0.50%)
Hematotoxicity	14(7.00%)	13(6.50%)	6 (3.00%)	2(1.00%)
Rash	21(10.50%)	15(7.50%)	7 (3.50%)	1(0.50%)
Vomiting	11(5.50%)	9(4.50%)	5 (2.50%)	0
constipation	1(0.50%)	2 (1.00%)	2 (1.00%)	0
headache	11(5.50%)	10(5.00%)	4 (2.00%)	1(0.50%)
Liver dysfunction	3(1.50%)	4(2.00%)	3 (1.50%)	0
Gastrointestinal hemorrhage	1(0.50%)	2(1.00%)	1 (0.50%)	0

## Discussion

Currently, cancer prognosis is typically determined by tumor burden and whether the patient presents cancer-related symptoms [15]. A significant proportion of HCC patients receive their diagnosis at an advanced stage, have inadequate hepatic reserve, or are ineligible for curative therapies due to tumor size, location, or metastasis [16–18]. Therefore, for patients with tumors, it is recommended that the optimal treatment strategy involves TACE, systemic treatment, or combined TACE therapy [7, 19, 20]. TACE treatment causes the production of higher levels of VEGF, with VEGF being the most potent among them. Therefore, the use of VEGF inhibitors in combination can significantly slow the progression of the tumor [21, 22]. Apatinib, a VEGFR-2 inhibitor that is selectively developed in China, has been approved for clinical use [10, 23, 24]. Several research reports suggest that combining TACE with Apatinib can considerably prolong the survival period of these patients [10, 25, 26]. However, not all patients who have advanced liver cancer benefit from this treatment method due to its heterogeneity. Hence, making clinical decisions for these patients necessitates careful consideration of various factors to ensure careful evaluation [11, 15]. Several prognostic models have been established to ascertain which patients benefit most from TACE [4, 12–14]. Nevertheless, these models are yet to be validated in TACE plus Apatinib treatment, resulting in the difficulty of identifying which patients can benefit significantly from this treatment. In this investigation, we evaluated the HAP, Six-and-Twelve, Up to Seven, and ALBI scoring models in 200 individuals with intermediate and advanced HCC who received TACE in combination with Apatinib medication.

Our research shows that the HAP, Six-and-Twelve, Up to Seven, and ALBI scoring systems displayed excellent discriminatory abilities in this cohort. The study revealed that the HAP score had a superior predictive capability, with a C-index significantly higher than that of Up to Seven and ALBI scores (*p* < 0.001). The Six-and-Twelve score demonstrated a smaller yet statistically significant difference in C-index compared to the HAP score (*p* < 0.05). The ALBI score is a weighted model that draws on albumin and bilirubin, and previous research has indicated that it accurately indicates hepatic functional reserve [27–29]. Both the Six-and-Twelve and Up to Seven scores place equal emphasis on the size and number of tumors. It is noteworthy that the ALBI score prioritises liver function, while the Six-and-Twelve and Up to Seven scores prioritise tumor load. Both of these factors are vital prognostic indicators for HCC. Multivariate analysis revealed that the ALBI score, tumor size, and PVTT are autonomous predictors of OS. PVTT frequently serves as a marker of tumor vascular invasiveness. This might lead to portal hypertension, compromising blood flow to liver tissue and influencing liver function, pointing to an unfavorable prognosis [30, 31]. However, the multivariate analysis did not identify tumor number as an independent risk factor for overall survival. This lack of significance may be attributed to the limited sample size of this study or the relatively short follow-up period, which could have led to biases in the statistical outcome.

The HAP score emphasizes four factors concerning hepatic functional reserve and tumor burden, which may explain its superior predictive performance. We employed the area under the AUROC curve concurrently to evaluate the predictive capabilities of the four scoring systems concerning short-term survival. The HAP score demonstrated commendable discriminatory abilities in predicting short-term survival at 6 months, 12 months, and 24 months. Interestingly, despite both the Six-and-Twelve and Up to Seven scores incorporating factors related to tumor burden, the C-index of the Six-and-Twelve score exceeds that of the Up to Seven score. We hypothesize that this might be due to the Six-and-Twelve score segregating patients into three subgroups, ultimately enhancing the granularity of the model. The C-index is a measure employed to appraise model performance, assessing the ability of the model torank different patients’ risks effectively. Dividing patients into three subgroups enables a more precise assessment of performance differences in the model’s ability to rank patient risk, potentially enhancing the C-index. It should be noted that previous research has indicated that the disparities between the Six-and-Twelve and Up to Seven scores may be linked to the use of original continuous variables in the calculation of the Six-and-Twelve score. In contrast, categorical data is used in the Up to Seven score, ultimately affecting performance evaluation [32].

In our study, we found that the median overall survival (OS) outcome was greater than that reported in previous research studies for intermediate and advanced hepatocellular carcinoma (HCC) patients who underwent TACE treatment alone (17.2 months vs. 10.7 months). Additionally, the median OS result mainly was in line with that reported in prior studies for intermediate and advanced HCC patients who underwent TACE in conjunction with Apatinib (17.2 months vs. 17 months) [33]. Hence, the outcomes of our study emphasize the probable benefits of the amalgamation of TACE with Apatinib to cure intermediate and advanced HCC patients. Importantly, in our research, over 60% of patients did not experience tumor metastasis, and over 60% of patients’ tumor size was less than 7 cm. This finding might be one justification for the marginally higher survival rate observed. Our study findings indicate that the combined use of TACE and Apatinib in treating intermediate and advanced HCC patients results in pronounced benefits from four scoring systems for prognostic stratification, with the HAP score demonstrating the highest degree of efficacy. This discovery highlights the importance of the HAP score in guiding treatment plans and predicting patient survival, offering solid backing and a point of reference for the clinical management of intermediate and advanced HCC patients. The use of immune checkpoint inhibitors in the treatment of hepatocellular carcinoma is expanding with the growing understanding of the immunological characteristics of the tumour microenvironment [34]. For patients with intermediate to advanced hepatocellular carcinoma, there are multiple systemic treatment options available. In the future, we can continue to explore the use of these scoring tools for predicting the effectiveness of different systemic treatment options [35].

Our research has some notable limitations. Firstly, it was a single-center retrospective design with a relatively small sample size, so we cannot ensure the broad applicability of the results. Additionally, we did not validate the predictive value of the four scoring systems in other medical centers, which may raise concerns about external validity. Given these limitations, we recommend conducting subsequent prospective multi-center studies to further validate our findings. These studies should involve larger sample sizes to increase statistical power and extend the external validity of the research. By validating across multiple medical centers, a more comprehensive assessment of the usability and reliability of the four scoring systems in intermediate and advanced HCC patients can be made.

## Conclusion

This research examined the predictive performance of four models, namely HAP, Six-and-Twelve, Up to seven, and ALBI scores, on survival rates of intermediate to advanced HCC patients who underwent TACE in combination with Apatinib. The HAP scoreexhibited the most reliable predictive ability, with Harrell’s C index producing the most accurate forecasts for survival outcomes. Thus, the HAP model is a useful and valuable tool for predicting the outcomes of intermediate and advanced HCC patients receiving TACE with Apatinib treatment. The appropriate staging system selection helps improve the management and assessment of these patients.

## Data Availability

Data used/ analyzed during the current study are available from the Corresponding Author on reasonable request.
